# Ozoralizumab, a Humanized Anti-TNFα NANOBODY^®^ Compound, Exhibits Efficacy Not Only at the Onset of Arthritis in a Human TNF Transgenic Mouse but Also During Secondary Failure of Administration of an Anti-TNFα IgG

**DOI:** 10.3389/fimmu.2022.853008

**Published:** 2022-02-22

**Authors:** Chihiro Ishiwatari-Ogata, Masanao Kyuuma, Hitoshi Ogata, Machi Yamakawa, Katsuya Iwata, Motoki Ochi, Miyuki Hori, Noriyuki Miyata, Yasuyuki Fujii

**Affiliations:** Research Headquarters, Taisho Pharmaceutical Co., Ltd., Saitama, Japan

**Keywords:** tumor necrosis factor, NANOBODY, VHH, rheumatoid arthritis, secondary failure, immunogenicity, anti-drug antibody, anti-TNFα antibody

## Abstract

Although the introduction of tumor necrosis factor (TNF) inhibitors represented a significant advance in the treatment of rheumatoid arthritis (RA), traditional anti-TNFα antibodies are somewhat immunogenic, and their use results in the formation of anti-drug antibodies (ADAs) and loss of efficacy (secondary failure). Ozoralizumab is a trivalent, bispecific NANOBODY^®^ compound that differs structurally from IgGs. In this study we investigated the suppressant effect of ozoralizumab and adalimumab, an anti-TNFα IgG, on arthritis and induction of ADAs in human TNF transgenic mice. Ozoralizumab markedly suppressed arthritis progression and did not induce ADAs during long-term administration. We also developed an animal model of secondary failure by repeatedly administering adalimumab and found that switching from adalimumab to ozoralizumab was followed by superior anti-arthritis efficacy in the secondary-failure animal model. Moreover, ozoralizumab did not form large immune complexes that might lead to ADA formation. The results of our studies suggest that ozoralizumab, which exhibited low immunogenicity in the animal model used and has a different antibody structure from that of IgGs, is a promising candidate for the treatment of RA patients not only at the onset of RA but also during secondary failure of anti-TNFα treatment.

## Introduction

Tumor necrosis factor alpha (TNFα) is a pleiotropic cytokine that has beneficial functions in immune regulation and host defense, but is also involved in a variety of inflammatory diseases, including rheumatoid arthritis (RA). RA is a common autoimmune disease that is characterized by chronic joint inflammation and progression to synovial hyperplasia and bone and cartilage destruction (1, 2). Since the first TNF inhibitor, a TNF-neutralizing agent, became available and incorporated into RA treatment protocols, TNF inhibitors have revolutionized the RA treatment paradigm (3–7). Five TNF inhibitors are now available for clinical use in RA, and all five are biological agents: etanercept, a TNF receptor fragment crystallizable (Fc) fusion protein (3); infliximab, an anti-TNFα chimeric monoclonal antibody (4); adalimumab and golimumab, anti-TNFα human monoclonal antibodies (5, 6); and certolizumab pegol, a pegylated anti-TNFα humanized antibody Fab’ fragment (7).

Anti-TNFα antibodies are often immunogenic during the treatment of human RA, and after repeated administration their immunogenicity results in the formation of anti-drug antibodies (ADAs). The therapeutic response to the biologics is favorable at the beginning of the intervention, but by neutralizing the function of the biologic or accelerating its clearance ADA formation results in loss of efficacy (secondary failure) (8). This is an important problem in the anti-TNFα antibody treatment of RA, because RA patients with a highly active immune system tend to be at high risk of ADA formation (9). ADA formation against adalimumab has been detected in 26%–44% and 17% of adalimumab-treated RA patients in Japan and Europe, respectively (10, 11). Administration of the immunosuppressant methotrexate (MTX) in combination with infliximab or adalimumab has been found to reduce ADA formation and results in a decrease in the incidence of secondary failure (12, 13), but since many elderly RA patients, especially in Asia, including in Japan, are unable to tolerate MTX (14), a new anti-TNFα antibody with low immunogenicity is needed for the treatment of RA.

Patients who fail to respond to the treatment with the first anti-TNFα antibody because of secondary failure are often switched to another anti-TNFα antibody, but the second anti-TNFα antibody does not always exert efficacy in all patients (15, 16). The ineffectiveness of the second anti-TNFα antibody is likely to be attributable to a structural relationship with the first anti-TNFα antibody and to the immunogenicity of the second anti-TNFα antibody itself (17). Since RA patients who display an immune response to the first biologic tend to develop ADAs to the second biologic, switching to a second structurally different anti-TNFα antibody with low immunogenicity should be effective in overcoming secondary failure of RA treatment. However, the effectiveness of switching to a structurally different anti-TNFα antibody in patients or animal models in secondary failure has never been demonstrated.

IgGs consist of two heavy chains and two light chains, but heavy-chain antibodies that are circulating in camelids such as llamas consist of only two heavy chains (18). NANOBODY^®^, the variable heavy chain domains of heavy-chain antibody (VHH), retains all of the antigen-binding activities of heavy-chain antibodies and possesses unique characteristics, including low molecular weight (12–15 kDa), rapid tissue penetration, and low immunogenicity (19, 20). New NANOBODY^®^ therapeutics superior to the IgGs therapeutics have recently been developed by taking advantage of these unique characteristics (21, 22).

The next-generation anti-TNFα antibody ozoralizumab is a 38 kDa humanized trivalent NANOBODY^®^ compound that consists of two anti-human TNFα NANOBODIES^®^ and an anti-human serum albumin (HSA) NANOBODY^®^. Ozoralizumab binds to two subunits of TNFα and should therefore potently neutralize its action (23–25). Ozoralizumab also binds to human and monkey serum albumin with similar dissociation constants (K_D_) of 24.4 nM and 24.1 nM, respectively, and its interaction with serum albumin results in a prolonged half-life (23, 25–27). A previous study showed that a surrogate anti-mouse TNFα NANOBODY^®^ compound of ozoralizumab was efficacious preventing RA in a murine collagen-induced arthritis model of RA (23). Ozoralizumab is expected to be more effective in circumventing secondary failure than traditional anti-TNFα antibodies because of its clearly different structure. However, the immunogenicity and neutralizing potency of ozoralizumab against human TNFα have never been elucidated.

Here we investigated the anti-arthritic efficacy of ozoralizumab in a human TNF transgenic mouse and attempted to predict clinical benefit of ozoralizumab for treatment of TNFα-induced arthritis. We also developed a new secondary-failure animal model and investigated whether switching treatment from the anti-TNFα IgG adalimumab to the anti-TNFα NANOBODY^®^ compound ozoralizumab would be effective in preventing secondary failure during continued treatment with adalimumab.

## Materials and Methods

### Generation of Ozoralizumab, A Trivalent Bispecific Construct Consisting of Two Anti-TNFα NANOBODIES^®^ and Anti-Human Serum Albumin NANOBODY^®^

Ozoralizumab is a 38 kDa humanized trivalent bispecific construct consisting of two anti-TNFα NANOBODIES^®^ and anti-HSA NANOBODY^®^ that was generated at Ablynx by a previously described method (23). Llamas were immunized with human TNFα and human muscle extract, which is rich in HSA, to induce the formation of anti-TNFα VHH and anti-HSA VHH. Both the anti-TNFα VHH and anti-HSA VHH were humanized by a complementary determining regions (CDR) grafting approach in which the CDR of the gene encoding llama VHH was grafted onto the most homologous human VHH framework sequence. Since binding to serum albumin prolongs the half-life of VHH (23, 26, 27), an anti-HSA VHH which efficiently binds murine serum albumin as well was incorporated into the two anti-TNFα VHHs. The three components were fused using a flexible Gly-Ser linker.

### *In Vitro* L929 Cell Cytotoxicity Assay

TNFα-sensitive mouse fibrosarcoma cells (L929 cells) were seeded in 96-well plates at a density of 5 × 10^3^ cells per well and grown for 48 hours as previously described (24, 28, 29). Various concentrations of ozoralizumab or clinical-glade adalimumab (Eisai, Japan) and 0.3 ng/mL human TNFα (FUJIFILM Wako Pure Chemical Corporation, Japan) were incubated in fresh Dulbecco’s Modified Eagle Medium (DMEM) for 15 minutes at room temperature and then added to designated wells containing L929 cells. The L929 cells were incubated for 24 hours at 37°C in this preincubated-medium with 1 μg/mL actinomycin D and then incubated for 10 minutes at room temperature with cell proliferation reagent CellTiter-Glo^®^ 2.0 (Promega Corporation, United States). Cell viability was measured by using the CellTiter-Glo assay according to the manufacturer’s protocol (30). The 50% inhibitory concentration (IC_50_) of each TNF inhibitor was determined by plotting titration curves in the XLfit software package (IDBS, United Kingdom).

### Mice

Tg197 human TNF transgenic mice (31) were bred and maintained in the animal facilities of Biomedcode Hellas SA under pathogen-free conditions. The animals were housed in standard plastic cages with wood chip bedding. The animal facility was maintained on an inverted 12/12-hour light/dark cycle at a constant room temperature of 22°C ± 2°C and approximately 60% relative humidity. Access to food pellets and filtered water were provided *ad libitum*. The test facility is accredited by the Hellenic Republic Directorate of Agricultural and Veterinary Policy (DAVP) of the Attica Region. All of the testing conditions conformed to the Presidential Decree applicable in Greece, which is the implementation of the EEC Directive 2010/63/EEC and approved by the DAVP of the Attica Region.

### Assessments of Chronic Polyarthritis Prevention and ADA Production

The Tg197 mouse is a well-established animal model of arthritis that can be used to investigate the pathogenesis and treatment of rheumatoid disease in humans (31, 32). Tg197 mice develop chronic polyarthritis with a 100% incidence starting at 3–4 weeks of age as a result of the overexpression of bioactive human TNF. The animal studies were conducted at Biomedcode Hellas SA, Greece. In the first study, 3-week-old mice were divided into 3 groups, each consisting of 10 heterozygous Tg197 male and female mice. The mean body weights and the genders of the animals were equally distributed among the 3 groups. The animals in the vehicle group were subcutaneously injected with phosphate-buffered saline (PBS) twice weekly from the start of the study at 3 weeks of age until euthanized at 11 weeks of age. The animals in the adalimumab group and ozoralizumab group were subcutaneously injected with 1 mg/kg anti-TNFα antibody twice weekly from the start of the study at 3 weeks of age until the end of the study at 16 weeks of age. Adalimumab (Abbvie) was purchased locally by Biomedcode Hellas SA. Serum samples were collected at 6, 8, 10 (all groups), 12, 14, and 16 (adalimumab group and ozoralizumab group) weeks of age. The animals in the vehicle group were euthanized at 11 weeks of age because of the increased mortality exhibited by the untreated Tg197 mice at that age.

The second study was conducted on 3-week-old Tg197 mice. The mice were divided into two groups: a vehicle group of 10 mice and an adalimumab group of 32 mice. The animals in the vehicle group were subcutaneously injected with PBS twice weekly until euthanized at 11 weeks of age, and the animals in the adalimumab group were subcutaneously injected with 1 mg/kg adalimumab twice weekly for 4 weeks. The 9 mice that had failed to generate ADAs against adalimumab at 6 weeks of age were eliminated from the study, and the ADA-positive animals in the adalimumab group were divided into two groups with equally distributed mean body weights and arthritis scores. The mice in one group continued to receive 1 mg/kg adalimumab (continued group, n = 13), and the mice in the other group were switched to administration of 1 mg/kg ozoralizumab (switched group, n = 10) at 7 weeks of age. The animals in the continued group and the switched group were subcutaneously injected with anti-TNFα antibody twice weekly until the end of the study at 12 weeks of age. The animals in the vehicle group were euthanized at 11 weeks of age because of the increased mortality exhibited by the untreated Tg197 mice at that age.

The analyses of ADAs against adalimumab or ozoralizumab and serum concentrations of anti-TNFα antibodies were performed 96 hours and 48 hours after antibody administration in the first study and the second study, respectively.

#### Macroscopic and Histopathological Evaluation

Macroscopic examinations of the mice for signs of arthritis were performed weekly from 3 weeks of age until the termination of the study. Each paw was scored on the following graded scale: 0, no disease; 0.5, mild disease; 1, mild to moderate disease; 1.5, moderate disease; 2, moderate to severe disease; 2.5, severe disease; 3, very severe disease. The severity of the histopathological signs of arthritis in both hind ankles was assessed in a blinded manner according to a standard histopathology scoring system as previously described (33, 34). For the histopathological assessment, all mice were euthanized 48 hours after the final dose, blood was then drawn by cardiac puncture, and both hind ankle joints of each mouse were collected for the histopathological evaluation. A control group of 3-week-old mice (n = 4) was used to assess baseline histopathological status. The ankle joints were fixed in 4% formaldehyde overnight at room temperature, and then demineralized in EDTA decalcification solution (13% EDTA in 0.1 M sodium phosphate buffer) at room temperature for 30 days and placed in PBS at 4°C till further processing. The samples were paraffin embedded in the sagittal plane, and the paraffin blocks were sectioned with a microtome. The sections were stained with H&E, and ankle histopathology was assessed by microscopic examination.

### Pharmacokinetic Study

Heterozygous 6-week-old Tg197 mice were gender-balanced and assigned to two groups for the pharmacokinetic study of adalimumab and ozoralizumab (35–37). The animals in the adalimumab group (n = 4) and the ozoralizumab group (n = 4) were subcutaneously injected with adalimumab or ozoralizumab both at 1 mg/kg. Blood samples were collected at 1, 6, 24, 48, 72, and 96 hours after administration of the anti-TNFα antibodies. Serum was isolated by centrifugation at 3,800 g at 4°C for 8 minutes, followed by transfer of the supernatants to tubes that were further centrifuged at 15,300 g at 4°C for 8 minutes. Adalimumab and ozoralizumab concentrations in the serum samples were measured by the enzyme-linked immunosorbent assays (ELISAs) described below, and the pharmacokinetic parameters of the anti-TNFα antibodies were calculated by using the WinNonlin version 8.0 software program (Certara, United States).

### Measurement of Serum Concentrations of Adalimumab and Ozoralizumab

Serum adalimumab and ozoralizumab concentrations were measured by ELISA (37). Immobilizer streptavidin F96 clear plates (Thermo Fisher Scientific, United States) were coated with 200 ng/mL biotinylated human TNFα for 1 hour at room temperature. The wells were washed with PBS containing 0.05% Tween 20 buffer (PBST) and then blocked for 1.5 to 3 hours at room temperature with PBST containing 1% bovine serum albumin (BSA). The serum samples of Tg197 mice in each study were 200-fold diluted with PBST containing 1% BSA. After washing the plates with PBST, the diluted serum samples were added to the wells, and the plates were allowed to stand overnight at 4°C. Adalimumab was detected with anti-human IgG-horseradish peroxidase (HRP) goat antibody (Jackson ImmunoResearch Laboratories, United States), and ozoralizumab was detected with anti-ozoralizumab rabbit antibody (Generated by Toray Research Center, Japan) followed by anti-rabbit IgG-HRP goat antibody (Bio-Rad Laboratories, United States). HRP activity was measured by using SureBlue™ 3,3’,5,5’-Tetramethylbenzidine (TMB) 1-Component Microwell Peroxidase Substrate (SeraCare Life Sciences, United States) according to the manufacturer’s protocol, and absorbance at 450 nm was measured with an ARVO X3 plate reader (PerkinElmer, United States). A standard curve was plotted for adalimumab and ozoralizumab in each plate in order to calculate the serum concentrations.

### ADA Detection

ADAs against adalimumab and ozoralizumab were measured by ELISA (37). The wells of 96-well microplates (Corning, United States) were coated with 1 μg/mL adalimumab or 500 ng/mL ozoralizumab overnight at room temperature and then washed with PBST. The wells were blocked for 1 hour at room temperature with PBST containing 4% BSA and then washed with PBST. Serum samples diluted 50-fold with PBST containing 1% BSA were added to the wells, and the plates were incubated for 1.5 hours at room temperature. After washing the wells with PBST, 3 μg/mL biotinylated adalimumab or 1 μg/mL biotinylated ozoralizumab was added to the wells, and the plates were incubated for 1.5 hours at room temperature. After washing the wells with PBST, HRP streptavidin (Vector Laboratories, United States) was added to the wells, and the plates were incubated for an additional 1.5 hours at room temperature. HRP activity was measured as described above in section *2.6 Measurement of serum concentrations of adalimumab and ozoralizumab.* Response ratios of the diluted serum samples were calculated as the ratio of the absorbance of each sample to that of the negative control (serum of non-treated mice). The serum samples were examined for the presence of ADAs by performing a screening assay. Serum samples whose response ratio was more than twice that of the negative control (cut-off point) were considered ADA-positive. The titers of the positive samples were calculated by using a titering assay. The maximum dilution factor of each ADA-positive serum sample was calculated as its relative titer.

### Neutralizing ADAs Detection

Neutralizing ADAs raised in the Tg197 mice injected with adalimumab or ozoralizumab were evaluated as follows. The wells of immobilizer streptavidin F96 clear plates (Thermo Fisher Scientific, United States) were coated with 200 ng/mL biotinylated human TNFα for 1 hour at room temperature. The wells were then washed with PBST and blocked for 1.5 to 3 hours at room temperature with PBS containing 1% BSA, and the wells were washed again with PBST. The serum samples were diluted 1,000-fold with PBS containing 1% BSA and mixed with 0.15 ng/mL anti-TNFα antibody (this concentration was equivalent to the approximately one thousandth of serum concentration of the mice 6–72 hours after a 1 mg/kg subcutaneous administration of anti-TNFα antibody). After pre-incubation for 90 minutes at room temperature, the mixtures were added to designated wells and incubated overnight at 4°C. The wells were then washed with PBST, and the amounts of active adalimumab and ozoralizumab were measured as described above in section *2.6 Measurement of serum concentrations of adalimumab and ozoralizumab*. The neutralizing effect of the serum was estimated based on the decrease in active anti-TNFα antibodies.

### Measurement of Bioactive Ozoralizumab in Mouse Serum by Performing an L929 Cell Cytotoxicity Assay

Bioactive anti-TNFα antibodies remaining in the serum samples of the Tg197 mice were measured by a TNFα-induced cytotoxicity assay using L929 mouse fibroblast cells. After incubating 0.03 ng/mL human TNFα and a 1,000-fold diluted serum sample from 12-week-old mice in the switched group in fresh DMEM for 15 minutes at room temperature, it was added to designated wells containing L929 cells (38). Cell seeding and a cell viability assay were performed as described above in section *2.2 In vitro L929 cell cytotoxicity assay*.

### Ouchterlony Double-Diffusion Assay

Formation of immune complexes by human TNFα and anti-TNFα antibodies was evaluated by an Ouchterlony double-diffusion assay. The central well of a rosette pattern of wells in an Ouchterlony gel plate (Thermo Fisher Scientific, United States) was loaded with 0.6 nmol human TNFα (39). Samples of anti-TNFα antibodies (0.6, 0.2, and 0.067 nmol) were applied to wells surrounding the center wells and allowed to diffuse overnight at 37°C under humid conditions. Protein precipitates formed by interactions between anti-TNFα antibodies and human TNFα were visualized in a bright field.

### Statistical Analysis

The body weight scores of the mice were analyzed parametrically by performing an ANOVA multiple comparison test and Dunnet’s *post-hoc* analysis. *In vivo* macroscopic and histopathological arthritis scores were compared by performing the Kruskal-Wallis test followed by Mann-Whitney *U*-test with Bonferroni correction. Tukey-Kramer multiple comparison tests were used to compare the data obtained for the neutralizing effects on anti-TNFα antibodies by ELISA and the cell-based assay.

## Results

### High TNFα-Neutralizing Potency of Ozoralizumab in the L929 Cell Cytotoxicity Assay

**Figure 1:**
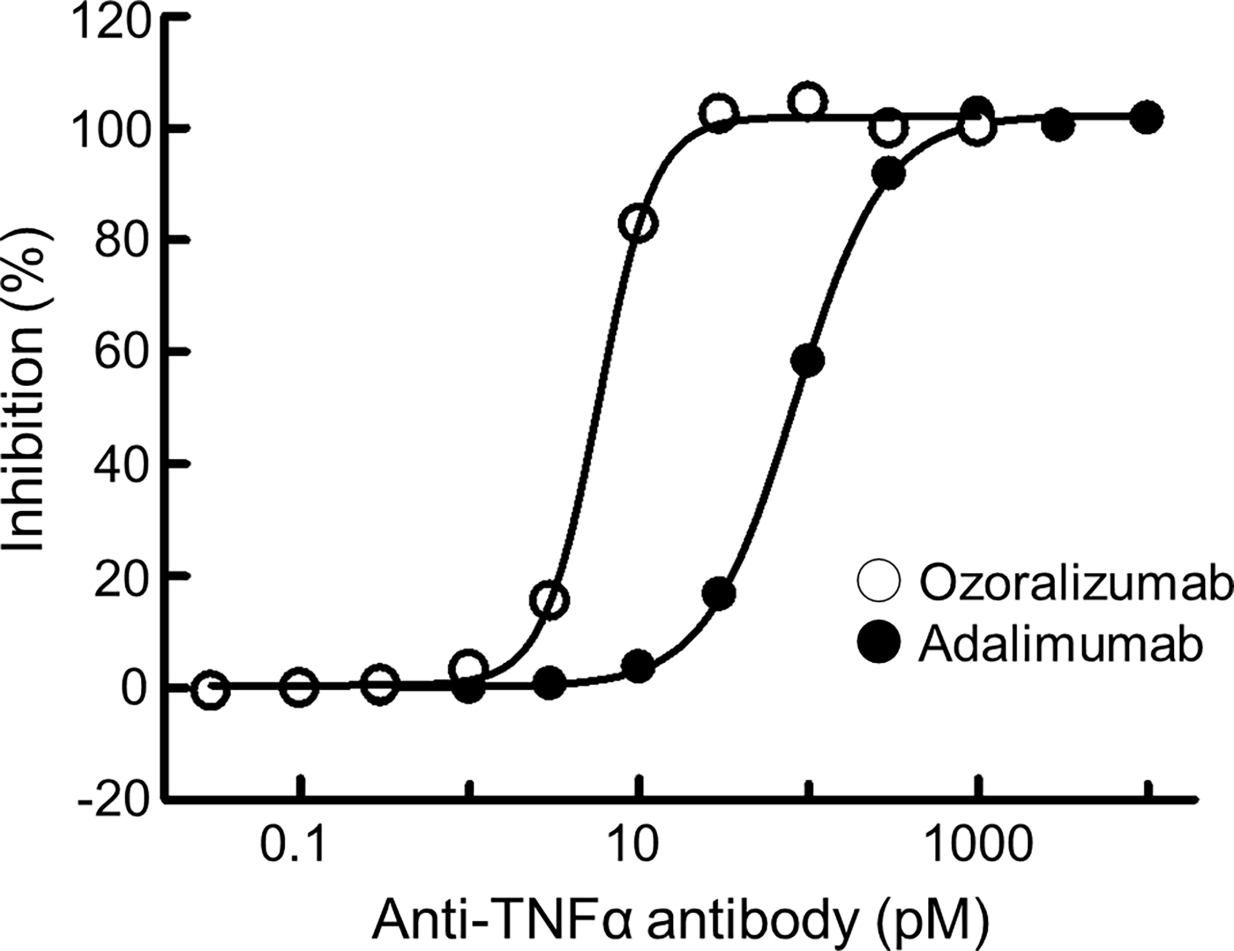
Neutralizing activity of ozoralizumab and adalimumab against human TNFα. Neutralizing activity was measured by the L929 cell cytotoxicity assay in the presence of a 0.3 ng/mL concentration of human TNFα. Inhibitory rates were calculated based on the assumption that cell survival was 0% when incubated with 0.3 ng/mL human TNFα and 100% when incubated without human TNFα. The IC_50_ values of ozoralizumab and adalimumab were 5.7 pM and 80.9 pM, respectively. The data are the means of values obtained in two experiments.

Ozoralizumab consists of two anti-TNFα NANOBODIES^®^ and one anti-HSA NANOBODY^®^. Hegen et al. found that human TNFα and monkey TNFα bind to ozoralizumab with very similar association and dissociation rates, resulting in nearly identical equivalent K_D_ values of 20.2 pM and 16.1 pM, respectively (Poster presented at the Annual Meeting of The American College of Rheumatology; November 6, 2011; McCormick Place Convention Center in Chicago). The L929 cell cytotoxicity assay is well known as a bioassay for TNFα and its biological inhibitors (24, 28, 29). The concentration of human TNFα used in this assay was 0.3 ng/mL and was chosen to yield nearly 80% cytotoxicity. Ozoralizumab neutralized human TNFα with an IC_50_ of 5.7 pM (**Figure 1**), whereas adalimumab neutralized human TNFα with an IC_50_ of 80.9 pM. These results indicate that the TNFα-neutralizing potency of ozoralizumab is superior to that of adalimumab in the L929 cell cytotoxicity assay.

### Low Immunogenicity and Long-Term Efficacy of Ozoralizumab in Comparison With Adalimumab in Tg197 Transgenic Mice

**Figure 2:**
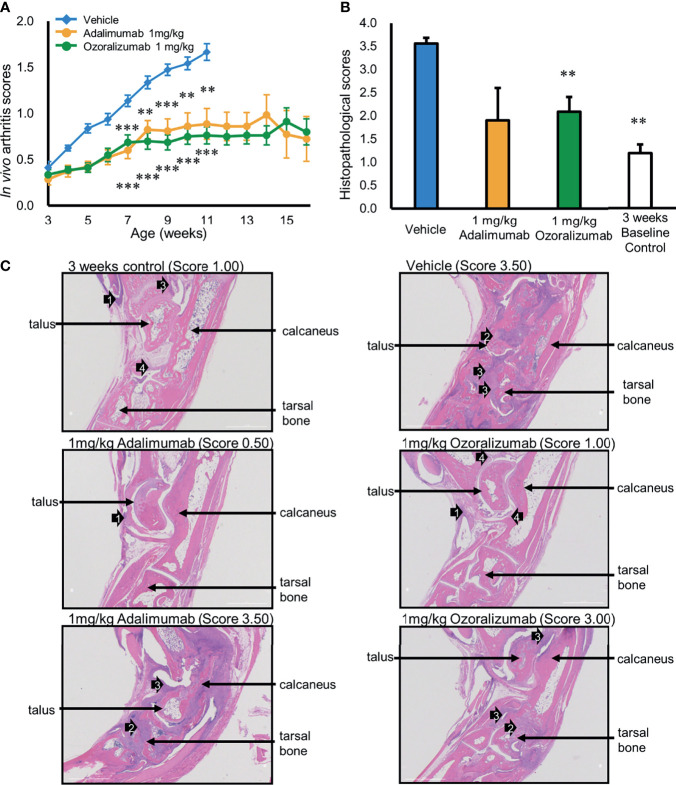
Efficacy of long-term administration of 1 mg/kg adalimumab and ozoralizumab twice weekly in Tg197 mice. **(A)** Changes in arthritis score. Arthritis scores were monitored weekly from 3 weeks of age to 16 weeks of age. Results are presented as the mean ± standard error (SEM) (n = 5–10). **p < 0.01 and ***p < 0.001 vs. vehicle (Mann-Whitney *U*-test with Bonferroni correction). **(B)** Means of arthritis histopathology scores at the end of the study: vehicle group at 11 weeks of age (n = 9), 1 mg/kg adalimumab group and 1 mg/kg ozoralizumab at 16 weeks of age (n = 5 and n = 10, respectively), and baseline control group at 3 weeks of age (n = 4). Results are shown as the mean ± SEM (n = 4–10). **p < 0.01 vs. vehicle (Mann-Whitney *U*-test with Bonferroni correction) **(C)** Representative histological images of the hind ankle joints. The arrows point to inflammatory cell infiltration (1), bone erosion (2), pannus (3), and synovial hyperplasia (4). Tissue sections of the ankle joints were obtained from the vehicle group of mice at 11 weeks of age, the anti-TNFα antibody group at 16 weeks of age, and the control group at 3 weeks. Original magnification, 25x.

ADA formation is considered one of the main reasons for the loss of therapeutic efficacy of anti-TNFα antibodies (8, 9). The Tg197 human TNF transgenic mice constitutively produced human TNFα, and then spontaneously developed severe arthritis (**Figure 2A**). We evaluated ADA formation against each of the anti-TNFα antibodies and the efficacy of long-term administration of these anti-TNFα antibodies against the TNFα-mediated polyarthritis in Tg197 mice. The anti-TNFα antibodies doses based on mice weight were adjusted considering the clinically used doses and the difference in body weight between humans and mice. In general, RA patients are injected 40 mg or 80 mg (0.6 mg/kg or 1.3 mg/kg for adult men weighing 60 kg) of adalimumab subcutaneously. In phase 3 clinical study of ozoralizumab, patients were injected 30 mg or 80 mg (0.5 mg/kg or 1.3 mg/kg for adult men weighing 60 kg) of ozoralizumab subcutaneously. Thus, the Tg197 mice were subcutaneously injected with a 1 mg/kg dose of ozoralizumab or adalimumab twice a week starting at 3 weeks of age. As shown in **Figure 2A**, both antibodies significantly and comparably reduced the arthritis scores compared with those in the vehicle group, indicating marked suppression of disease progression (ozoralizumab group: 0.76, p < 0.001; adalimumab group: 0.89, p < 0.01, vs. vehicle in both groups). There was premature mortality in the vehicle group of Tg197 mice starting at 9 weeks of age (**Supplementary Figure 1A**). Premature mortality also occurred in the adalimumab group starting at 11 weeks of age, and its incidence reached 50% at 16 weeks of age. Importantly, no premature mortality was observed in the ozoralizumab group, at least up to 16 weeks of age. Body weight increase was impaired in the vehicle group, but there was a sustained increase in body weight in the ozoralizumab group and adalimumab group (**Supplementary Figure 1B**).

Joint damage was assessed histopathologically in the hind paws of the Tg197 mice in the vehicle group, ozoralizumab group, adalimumab group, and 3-week-old control group. The synovial lining of the Tg197 mice of vehicle group became markedly thickened because of synovial cell proliferation and inflammatory cell infiltration, and this proliferative mass, the pannus, invaded and progressively destroyed articular cartilage and bone, leading to irreversible destruction of joint structure and function. The histological changes characteristic of chronic inflammatory arthritis in the hind paws of the Tg197 mice were first noted at 3 weeks of age. Importantly, there was a significant reduction (p < 0.01) in the mean histopathologic score in the Tg197 mice that had been treated with ozoralizumab for 13 weeks in comparison with the vehicle group (**Figures 2B, C**).

A link between the presence of ADAs and both decreased serum drug concentrations and clinical efficacy of anti-TNFα antibodies has been well documented (8, 9). ADA titers have been reported to be associated with low levels or an absence of anti-TNFα antibodies in serum samples and to result in a weakened therapeutic response and even exacerbation of the underlying disease (40). To investigate whether ADAs reflect reductions in the serum levels of antibodies administered and result in a compromised therapeutic response, we measured ADA levels in serum samples obtained from Tg197 mice after administration of anti-TNFα antibodies. ADA formation against each of the anti-TNFα antibodies in the groups treated with ozoralizumab or adalimumab twice a week was monitored every two weeks until 16 weeks of age (**Tables 1A, B**). ADAs against adalimumab were detected in the serum obtained at 6 weeks of age, and the ADA level (log titer) rose dramatically, reaching an 80% incidence in the 16-week-old mice in the adalimumab group. Remarkably, ADAs against ozoralizumab were detected in hardly any of the mice in the ozoralizumab group, at least up to 13 weeks after the start of administration. Taken together, our findings demonstrate that ozoralizumab has strong ability to neutralize TNFα activity, without being immunogenic, during long-term administration to Tg197 mice.

**Table 1:** Detection of ADAs against adalimumab **(A)** and ozoralizumab **(B)** in the serum of the mice injected with a 1 mg/kg dose of the anti-TNFα antibodies twice a week.

A							
Group	ADAs against adalimumab (Log titer)						
	6 weeks	8 weeks	10 weeks		12 weeks	14 weeks	16 weeks
Adalimumab group	Neg	Neg	Neg		Neg	Neg	Neg
	Pos (1.43)	Pos (2.86)	Pos (3.34)		Pos (3.34)	N.S.	N.S.
	Neg	Neg	Neg		Neg	Neg	Neg
	Pos (0.954)	Pos (2.39)	Pos (2.86)		Pos (3.34)	Pos (3.34)	Pos (3.34)
	Pos (1.43)	Pos (2.86)	Pos (3.34)		Pos (3.82)	Pos (3.82)	Pos (3.82)
	Pos (1.43)	Pos (2.86)	Pos (2.86)		Pos (2.86)	Pos (2.86)	Pos (2.86)
	Pos (1.91)	Pos (2.86)	Pos (3.34)		Pos (3.82)	N.S.	N.S.
	Pos (1.43)	Pos (2.86)	Pos (3.34)		Pos (3.82)	Pos (3.82)	N.S.
	Pos (2.86)	Pos (3.34)	Pos (3.34)		Pos (3.34)	Pos (3.34)	N.S.
	Pos (1.91)	Pos (2.86)	Pos (3.82)		N.S.	N.S.	N.S.
**B**							
**Group**	**ADAs against ozoralizumab (Log titer)**						
	**6 weeks**	**8 weeks**	**10 weeks**		**12 weeks**	**14 weeks**	**16 weeks**
Ozoralizumab group	Neg	Neg	Neg		Neg	Neg	Neg
	Neg	Neg	Neg		Neg	Neg	Neg
	Neg	Neg	Neg		Neg	Neg	Neg
	Neg	Neg	Neg		Neg	Neg	Neg
	Neg	Neg	Neg		Neg	Neg	Neg
	Neg	Neg	Neg		Neg	Neg	Neg
	Neg	Neg	Neg		Neg	Neg	Neg
	Neg	Neg	Neg		Neg	Neg	Neg
	Neg	Pos (<0.477)	Neg		Neg	Neg	Neg
	Neg	Neg	Neg		Neg	Neg	Neg

Pos, positive; Neg, negative (not above the screening cut-off point); N.S., no sample.

**Figure 3:**
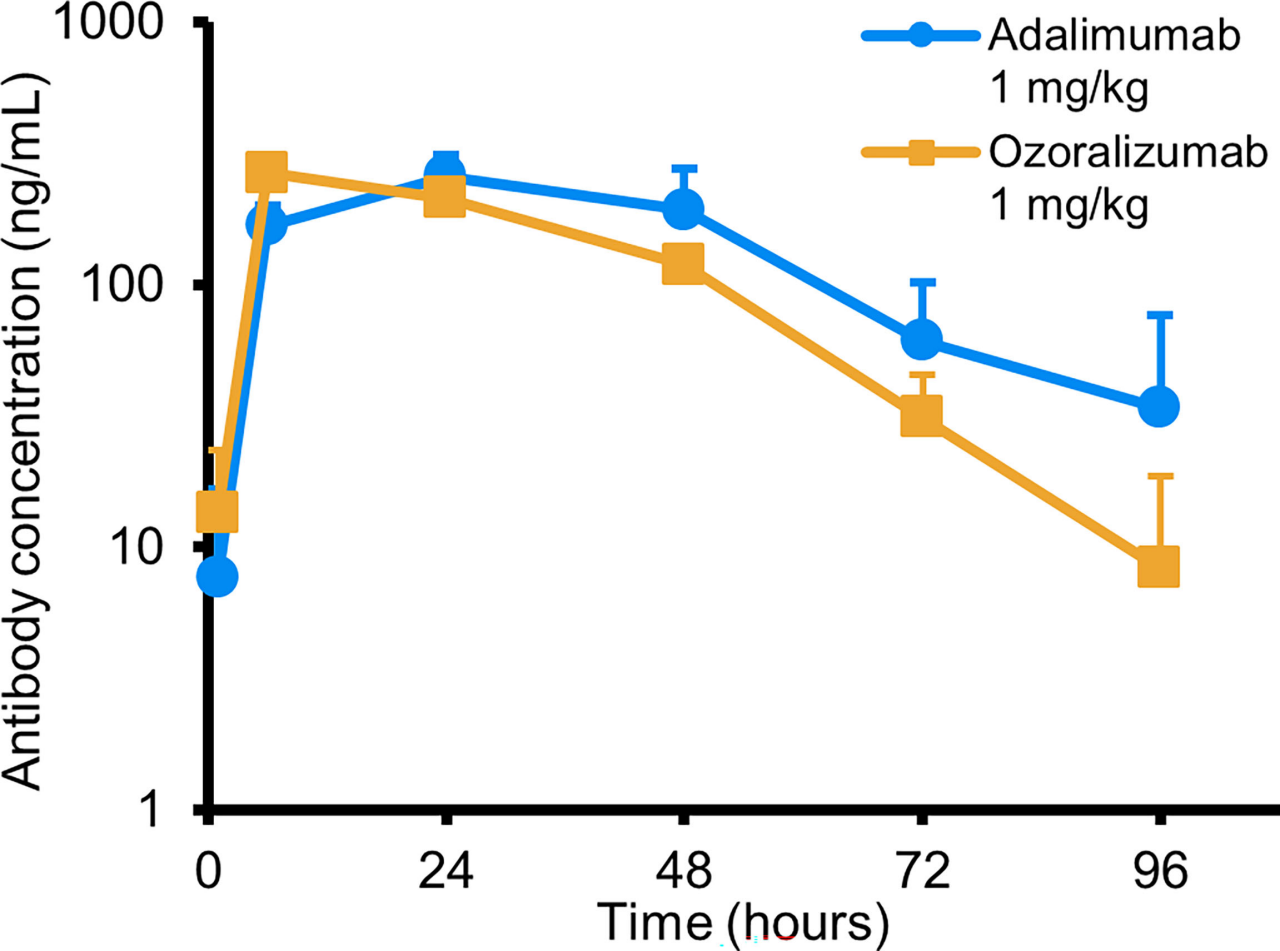
Serum adalimumab and ozoralizumab concentrations of the Tg197 mice following a single subcutaneous administration. Serum samples were collected 1, 6, 24, 48, 72, and 96 hours after administration. Each time point represents the mean ± standard deviation (SD) (n = 4).

To exclude a possible difference in pharmacokinetics between ozoralizumab and adalimumab, we monitored the serum concentrations of these antibodies in Tg197 mice after a single subcutaneous dose of 1 mg/kg of ozoralizumab or adalimumab. The results showed comparable serum concentration-time curves for ozoralizumab and adalimumab (**Figure 3**). The pharmacokinetic parameters obtained for ozoralizumab and adalimumab were: peak concentration (C_max_), 265 ng/mL and 257 ng/mL, respectively; time to peak concentration (t_max_), 6 hours and 24 hours, respectively; and elimination half-life (t_1/2_), 12 hours and 19 hours, respectively. Moreover, after twice weekly administration of adalimumab, the serum adalimumab concentrations of the Tg197 mice at 16 weeks of age were below the lower limit of quantification (<10 ng/mL, data not shown), indicating that the emergence of ADAs can cause serum drug levels to drop to sub-therapeutic levels.

### Efficacy of Switching the Treatment of Arthritis in the Tg197 Mice From Adalimumab to Ozoralizumab

**Figure 4:**
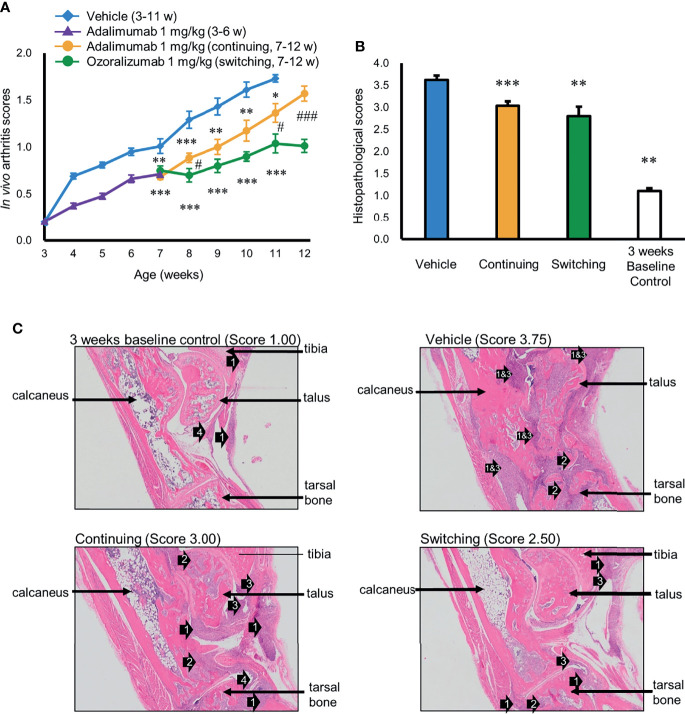
Prevention of polyarthritis in Tg197 mice by switching from adalimumab to ozoralizumab. The mice that were positive for adalimumab-induced ADA at 6 weeks of age were treated with either adalimumab or ozoralizumab twice a week from 7 weeks of age onward. **(A)** Changes in arthritis score. Arthritis scores were monitored weekly from 3 weeks of age to 11 or 12 weeks of age. Results are expressed as the mean ± SEM (n = 10–32). *p < 0.05, **p < 0.01 and ***p < 0.001 vs. vehicle and ^#^p < 0.05 and ^###^p < 0.001for the continued group vs. the switched group (Mann-Whitney *U*-test with Bonferroni correction). **(B)** Mean arthritis histopathology scores at the end of the experiments: vehicle group at 11 weeks of age, continued group and switched group at 12 weeks of age, and baseline control group at 3 weeks of age. Results shown are the mean ± SEM (n = 4–10). **p < 0.01 and ***p < 0.001 vs. vehicle (Mann-Whitney *U*-test with Bonferroni correction) **(C)** Representative histological images of the hind ankle joints of the Tg197 mice at the end of the study. The arrows point to inflammatory cell infiltration (1), bone erosion (2), pannus (3), and synovial hyperplasia (4). Tissue sections of the ankle joints were obtained from the vehicle group at 11 weeks of age, continued group and switched group at 12 weeks of age, and control group at 3 weeks of age. Original magnification, 25x.

Ozoralizumab has a unique structure that differs from the structure of IgG-type antibodies and fails to induce ADAs after repeated administration. We investigated whether ozoralizumab is capable of circumventing ADA-induced secondary failure after repeated administration of adalimumab by switching from administration of adalimumab to ozoralizumab in Tg197 transgenic mice. ADAs against adalimumab were detected in 23 of 32 mice treated with adalimumab during the first 3 weeks, and the log titers of ADA against adalimumab in the 6-week-old mice ranged from <0.477 to 1.91 (data not shown). The mice that were positive for adalimumab-induced ADAs at 6 weeks of age were divided into 2 groups: a continued group, in which adalimumab administration was continued for an additional 6 weeks, and a switched group, in which adalimumab was switched to ozoralizumab administration for the next 6 weeks. The *in vivo* arthritis scores of the mice in the vehicle group increased rapidly from 3 weeks of age onward and reached 1.73 at 11 weeks of age (**Figure 4A**). At 11 weeks of age, the arthritis scores of both the continued group and switched group were significantly lower than in the vehicle group (**Figure 4A**). The increases in histopathological scores were also significantly lower in both the continued group and switched group in comparison with the scores in the vehicle group (**Figures 4B, C**). Importantly, the arthritis scores of the switched group were much lower than in the continued group at 11 weeks of age and 12 weeks of age (1.04 vs. 1.36, p < 0.01 and 1.01 vs. 1.57, p < 0.001, respectively), indicating that switching to ozoralizumab markedly suppressed disease progression in comparison with continued administration of adalimumab. Furthermore, at 8 weeks of age, one week after the switching to ozoralizumab, the arthritis scores were already lower in the switched group compared to the continued group (0.70 vs. 0.84, p < 0.05), indicating rapid suppression of disease progression within a short period. It is also noteworthy that throughout the period of administration of ozoralizumab the arthritis scores in the switched group were steadily suppressed, whereas the arthritis scores in the continued group gradually and progressively increased until the end of the study.

### Ozoralizumab Is Not Neutralized by Adalimumab-Induced ADAs

**Figure 5:**
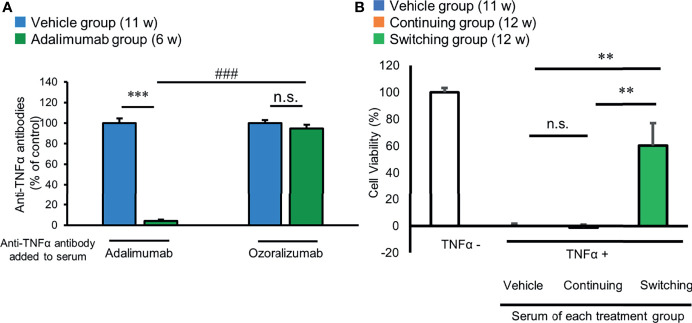
Neutralizing activity of adalimumab-induced ADAs. **(A)** Neutralizing activity of the sera from the adalimumab group of mice against exogenously added adalimumab or ozoralizumab was measured by ELISA. The serum samples obtained from 11-week-old mice in the vehicle group were used as a control. Results are expressed as the mean ± SEM (n = 8–21). n.s., not significant; ***p < 0.001; serum of the adalimumab group of mice vs. serum of the vehicle group of mice. ^###^p < 0.001; adalimumab vs. ozoralizumab in the serum of the adalimumab group of 6-week-old mice (Tukey’s test). **(B)** The remaining TNFα inhibitory effect of circulating anti-TNFα antibodies in the mice sera from the second study was measured by cell cytotoxicity assay. TNFα cell viability test with serum collected from the continued group and switched groups. The sera were collected from the vehicle group of mice and the continued group and switched group at the end of the efficacy study. Serum protection of cell viability was tested in the TNFα-induced cytotoxicity assay using cell line L929. Recombinant human TNFα (0.03 ng/mL) and actinomycin D (1 μg/mL) were used in all experiments. The data are percentages of cell viability when the results in the absence of addition of exogenous human TNFα were assumed to be 100%. The results are expressed as the mean ± SEM (n = 8–13). **p < 0.01 (Turkey’s test).

We next investigated whether ozoralizumab is capable of exerting neutralizing activity even after ADA formation induced by repeated administration of adalimumab. To do so, we tested the neutralizing activity of the serum containing adalimumab-induced ADAs against exogenously added anti-TNFα antibodies by ELISA. In this experiment, serum samples collected from the vehicle group and adalimumab group of mice at 6 weeks of age were incubated with exogenously added adalimumab or ozoralizumab, and then the remaining binding activity of the anti-TNFα antibodies to immobilized TNFαwas measured by ELISA (**Figure 5A**). The TNFα-binding activity of adalimumab and ozoralizumab was unaffected by the serum from the vehicle group, whereas the remaining TNFα-binding activity of the adalimumab added to the serum containing ADAs against adalimumab was almost negligible (4.3%), indicating that most of the adalimumab was neutralized by the ADAs. By contrast, 94.8% of the TNFα-binding activity of ozoralizumab remained in the serum under the same conditions, demonstrating that ADAs against adalimumab do not neutralize ozoralizumab.

We further confirmed the difference in inhibitory effect on the cytotoxicity of circulating anti-TNFα antibodies in the serum samples obtained from the continued group and switched group of 12-week-old mice. The serum samples of the continued group failed to suppress cytotoxicity induced by human TNFα, cell viability was significantly increased in the serum samples from the switched group, (**Figure 5B**). The results thus demonstrated that the ozoralizumab in the serum of the switched group retained its potency to inhibit TNFα activity and that the serum of the mice that had received repeated administrations of adalimumab had lost its inhibitory activity against TNFα.

### Negligible ADA Formation Against Ozoralizumab in the Mice With Adalimumab-Induced ADAs

It was important to determine whether, ozoralizumab is effective in suppressing TNFα-induced arthritis once ADAs have generated after repeated administration of adalimumab. To do so, we investigated ADA levels against adalimumab and ozoralizumab in the mice in the continued group and switched group at the end of the study (**Table 2**). ADAs against adalimumab were detected in the switched group as well as the continued group even though adalimumab administration had been discontinued in the switched group during the final 6 weeks (**Table 2A**). Remarkably, no ADAs against ozoralizumab were detected in most of the mice in the switched group (8 out of 10, **Table 2B**), even though ADAs against adalimumab remained high in the serum of these mice at the end of study. The log titer of the ADAs against ozoralizumab detected in the switched group was almost negligible (<0.477, **Table 2B**). Thus, even though ozoralizumab was administered to the mice that had generated ADAs against adalimumab, ADAs were not generated against ozoralizumab, and as a result switching to ozoralizumab was capable of continuing to suppress the arthritis. Furthermore, the serum adalimumab concentrations in most of the continued group were below the lower limit of quantification (<10 ng/mL, **Table 2A**). However, the serum concentration of ozoralizumab in the switched group was 12.1–1690 ng/mL (**Table 2B**), consistent with the marked difference in ADA formation in response to adalimumab and ozoralizumab.

**Table 2:** ADAs and serum concentrations of adalimumab **(A)** and ozoralizumab **(B)** in the continued group and the switched group at the end of the study.

A		
Group	ADAs against adalimumab (Log titer)	Serum adalimumab concentration (ng/ml)
Continued group	Pos (2.86)	BLQ
	Pos (3.34)	BLQ
	Pos (2.39)	BLQ
	Pos (0.477)	10.9
	Pos (1.43)	BLQ
	Pos (2.39)	BLQ
	Pos (2.86)	BLQ
	Pos (2.86)	BLQ
	Pos (3.34)	BLQ
	Neg	90.5
	Pos (2.86)	BLQ
	Pos (2.86)	BLQ
	Pos (3.34)	BLQ
Switched group	Pos (0.954)	BLQ
	Pos (2.39)	BLQ
	Pos (1.43)	BLQ
	Pos (1.91)	BLQ
	Pos (1.91)	BLQ
	Pos (1.91)	BLQ
	Pos (1.91)	BLQ
	Pos (1.91)	BLQ
	Pos (1.43)	BLQ
	Pos (1.91)	BLQ
**B**		
Group	ADAs against ozoralizumab (Log titer)	Serum ozoralizumab concentration (ng/ml)
Switched group	Neg	538
	Neg	170
	Neg	1690
	Neg	101
	Neg	15.4
	Pos (<0.477)	BLQ
	Neg	161
	Neg	12.1
	Neg	14.1
	Pos (<0.477)	BLQ

Pos, positive; Neg, negative (not above the screening cut-off point); BLQ, below the lower limit of quantification (<10.0 ng/mL).

### Difference in Immune Complex Formation Between Ozoralizumab and Adalimumab

**Figure 6:**
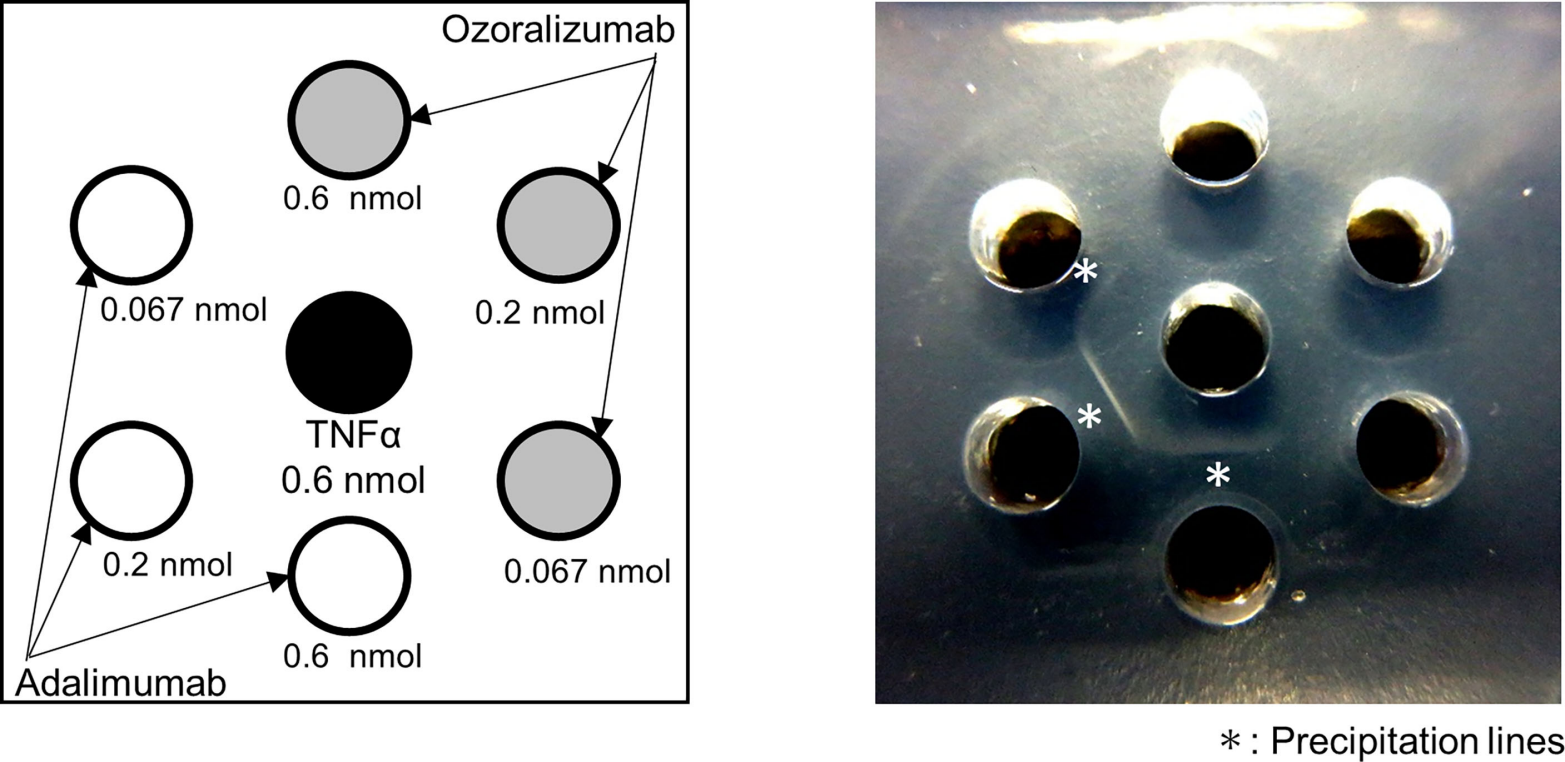
Precipitation of protein complexes formed by anti-TNFα antibodies and human recombinant TNFα. Samples were applied to proper rosette wells of Ouchterlony agarose gels with 0.6 nmol of TNFα in the center wells and anti-TNFα antibodies in the outside wells. Adalimumab and ozoralizumab were applied to wells at molar ratios of 3:1, 1:1, and 1:3 (0.6, 0.2, and 0.067 nmol) to TNFα trimer (0.2 nmol). The left panel illustrates the procedure for filling the wells with TNFα and anti-TNFα antibodies, and the right panel shows the results of interactions between the anti-TNFα antibodies and TNFα. Precipitation lines are marked with an asterisk.

Formation of a large immune complex has been reported to be critical for developing high immunogenicity (9). IgG-type antibodies can form a very large complex by strongly interacting with two different trimeric TNFα molecules (41). To investigate whether ozoralizumab also forms a large complex, we performed the Ouchterlony double-diffusion assays with different antibody to TNFα concentration ratios. Adalimumab formed a large enough immune complex with TNFα to be detected as a precipitable line in the assay, whereas no precipitation line was observed with ozoralizumab in agarose gels, suggesting that it does not form a large immune complex with TNFα (**Figure 6**).

The results showed that ozoralizumab does not form a large immune complex that might lead to ADA formation, and suggest that this difference is one of the reasons that ozoralizumab is functionally distinct from adalimumab.

## Discussion

**Figure 7:**
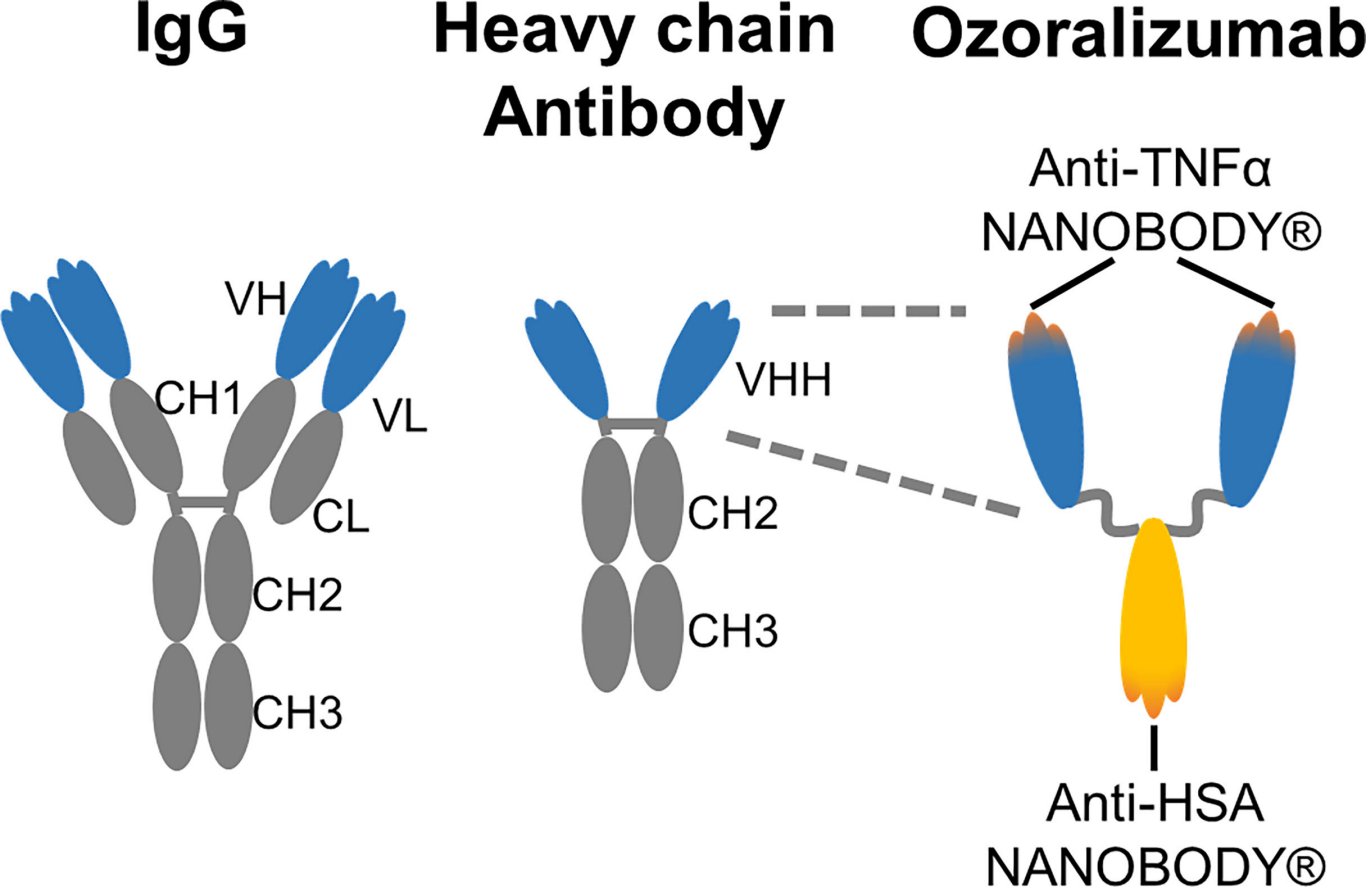
Schematic representation of IgG, camelid heavy chain antibody and ozoralizumab.

The introduction of biological TNF inhibitors was a significant advance in the management of RA (3–7). Despite this advance, the available TNF inhibitors elicit some unavoidable aversive immune responses and frequently lose the efficacy during long-term treatment (8). Ozoralizumab is a trivalent humanized low molecular weight antibody consisting of two anti-TNFα NANOBODIES^®^ and one anti-HSA NANOBODY^®^ that was developed as a next-generation anti-TNFα humanized antibody (**Figure 7**). The bivalent anti-TNFα NANOBODY^®^ of ozoralizumab binds to the two TNFα molecules and potently neutralizes the activity of TNFα (23–25).

In these studies reported here we demonstrated that ozoralizumab potently suppressed arthritis progression and without inducing ADA formation during long-term administration. One of the problems with anti-TNFα antibody therapeutics is secondary failure due to ADA formation, especially in the patients with inflammatory diseases like RA. ADAs can be classified into 2 types, neutralizing ADAs and non-neutralizing ADAs (17, 42). Neutralizing ADAs bind to the antigen-recognition site of the antibody and inhibit its pharmacological function. Non-neutralizing ADAs reduce the systemic distribution of the antibody by increasing the rate of antibody clearance. A study has reported finding that 50.2% of patients treated with adalimumab were ADA-positive and that half of the ADAs corresponded to neutralizing ADAs (43). In our present studies, we developed an animal model of secondary failure of adalimumab, the most widely used anti-TNFα antibody, by using Tg197 mice, which exhibits pathology that closely resembling human RA. ADAs were induced in 80% of the mice in the adalimumab group, and the serum adalimumab concentrations were below the lower limit of quantification, indicating accelerated clearance of adalimumab. Moreover, exogenously added adalimumab was significantly captured by ADAs in the serum of adalimumab-treated mice and failed to bind to TNFα, indicating that neutralizing activity of ADAs against adalimumab is also induced in Tg197 transgenic mice in response to adalimumab administration. These results are consistent with the findings reported in patients with secondary failure to anti-TNFα antibody (40). Naïve Tg197 transgenic mice and mice that generated adalimumab-induced ADA have both been shown to exhibit progressive weight loss, and they died or were euthanized before completion of the experimental protocol because of severe arthritis and cachexia (31). The arthritis and histopathological damage in these mice thus seemed to be more severe than the arthritis and histopathological scores calculated from data obtained in the surviving mice in our experiment. Thus, the severity of the arthritis of adalimumab group in our study may even have been underestimated. In contrast, ozoralizumab neither induced ADAs during long-term administration nor elicited early mortality in the Tg197 transgenic mice in our study. Thus, ozoralizumab provides a superior anti-TNFα antibody by maintaining low immunogenicity and long-term efficacy.

RA patients who have become resistant to treatment with an anti-TNFα antibody as a result of secondary failure are often switched to treatment with another anti-TNFα antibody (8). However, a number of such patients fail to respond to the second anti-TNFα antibody as well (44, 45). The ineffectiveness of the second antibody is most likely attributable to the ADAs produced during treatment with the first antibody because of a structural similarity between the first and second antibodies and its own immunogenicity (8, 9). Patients with ADAs against infliximab have been reported to more frequently generate ADAs against adalimumab than infliximab-ADA-free patients or anti-TNFα antibody-untreated patients (45). Non-neutralizing ADAs may affect the reactivity of the second anti-TNFα antibody, because the first and second antibodies have a common constant region, and thus they may reduce the systemic distribution of the antibody by increasing its clearance rate (42, 46, 47). Because of its protein polymorphisms the constant region of IgG induces non-neutralizing ADAs when injected into incompatible patients (46, 47). Since ozoralizumab consists of three NANOBODIES^®^ that are structurally different from such IgGs as infliximab and adalimumab and lacks the constant region, the ADAs against constant region of pretreated IgG should not bind to ozoralizumab. Furthermore, ozoralizumab per se had lower immunogenicity than adalimumab, and, importantly, did not induce ADA formation in the mice that were positive for adalimumab-induced ADAs. Hence, switching to ozoralizumab provided anti-arthritis efficacy in the Tg197 mice that was superior to continued administration of adalimumab. Ozoralizumab may be desirable for use as a TNF inhibitor not only for the initial TNF inhibitor treatment of RA patients but also as the second TNF inhibitor in ADA-positive RA patients.

Accumulating evidence indicates that the formation of a large immune complex is critical to developing high immunogenicity (9, 41). Ozoralizumab did not form a large immune complex that might lead to ADA formation. IgGs like adalimumab form a large immune complex that is stable (41). The large immune complex in turn facilitates uptake of the complex by antigen-presenting cells, and it is cleared from the circulation more rapidly than small complexes (9, 37, 48). Ozoralizumab is constructed from three small NANOBODIES^®^ fused together with a flexible linker (24, 25). Hence, the molecular weight of the NANOBODY^®^ compound-TNFα complex is lower than that of IgG-TNFα complexes (24, 41). The flexible geometry of the short linker and the small size of the NANOBODY^®^ binding site allow these bivalent NANOBODIES^®^ to bind to the two epitopes on the surface of a TNFα trimer molecule in the same way. Since ozoralizumab does not form a large immune complex, it has low immunogenicity and is cleared normally.

Clinically, some TNF inhibitors, including adalimumab, are prescribed for RA patients in combination with the immunosuppressant MTX to prevent the formation of ADAs (12, 13), but a number of patients are unable to tolerate MTX because of its side effects (14). The Fc region of IgGs is critical for the antigen presentation required to produce antibodies and for immune complex clearance by interacting with FcγRs on phagocytic cells (49). FcγR-mediated pathways enhance macrophage polarization and the functional activity of antigen-presenting cells, including their capacity for antigen processing and presentation, as well as regulate the maturation and activation of dendritic cells (50), and MTX is used to suppress the function of FcγRs (51). NANOBODIES^®^ lack the Fc region of IgGs. Chloé et al. reported observing the absence of significant activation of dendritic cells upon exposure to NANOBODIES^®^ and a low capacity of NANOBODIES^®^ to induce dendritic cell-mediated T-cell proliferation (52). In our own study, ozoralizumab did not induce ADA formation during long-term administration in the absence of combined administration of MTX, and it effectively suppressed the progression of arthritis in Tg197 mice. Ozoralizumab could be freed from the effects of the FcγR function, i.e., from activation of antigen-presenting cells. Therefore, ozoralizumab is expected to be usable in the patients showing MTX intolerance.

In summary, human TNF-expressing transgenic mice were used to assess the therapeutic usefulness of ozoralizumab as a TNF inhibitor, and the results of our first study indicated that ozoralizumab is a superior TNF inhibitor that exhibits lower immunogenicity and long-term efficacy in Tg197 mice in comparison with a traditional anti-TNFα antibody. Moreover, switching to ozoralizumab under secondary-failure conditions suppressed arthritis progression, because no ADAs against ozoralizumab were generated. Taken together, the results of our studies suggest that ozoralizumab is a promising candidate for the treatment of RA patients not only at the onset of RA but during secondary failure of anti-TNFα treatment as well.

## Data Availability Statement

The raw data supporting the conclusions of this article will be made available by the authors, without undue reservation.

## Ethics Statement

The animal study was reviewed and approved by the Directorate of Agricultural and Veterinary Policy (DAVP) of the Attica Region.

## Author Contributions

The experiments and data analyses were performed by CI-O, MK, HO, MY, KI, and MO. The experiments were designed and supervised by MH, NM, and YF. The manuscript was written by CI and edited by MK, HO, MY, KI, MO, MH, NM, and YF. All authors contributed to the article and approved the version submitted.

## Funding

This work was supported by Taisho Pharmaceutical Co., Ltd.

## Conflict of Interest

Author CI-O, MK, HO, MY, KI, MO, MH, NM, and YF are employed by Taisho Pharmaceutical Co., Ltd. This study received funding from Taisho Pharmaceutical Co., Ltd. The funder had the following involvement with the study: the study design, collection, analysis, interpretation of data, the writing of this article or the decision to submit it for publication.

## Publisher’s Note

All claims expressed in this article are solely those of the authors and do not necessarily represent those of their affiliated organizations, or those of the publisher, the editors and the reviewers. Any product that may be evaluated in this article, or claim that may be made by its manufacturer, is not guaranteed or endorsed by the publisher.
