# Supplementary material for: Ozoralizumab, a Humanized Anti-TNFα NANOBODY® Compound, Exhibits Efficacy Not Only at the Onset of Arthritis in a Human TNF Transgenic Mouse but Also During Secondary Failure of Administration of an Anti-TNFα IgG
Source: Front Immunol. 2022 Feb 22;13:853008. doi: 10.3389/fimmu.2022.853008 (PMC8902368; doi:10.3389/fimmu.2022.853008)
Supplement: Supplementary file 1 [file DataSheet_1.pdf]

Supplementary Material

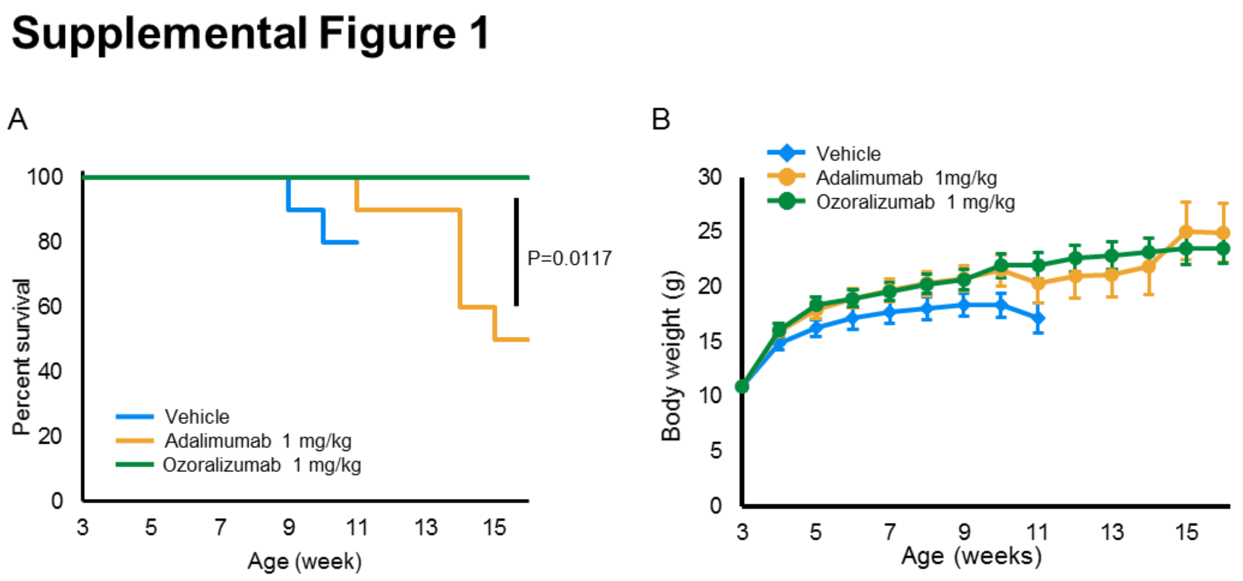

Supplementary Figure 1. Mortality and body weight changes during long-term administration of 1 mg/kg doses of adalimumab and ozoralizumab. **(A)** Survival rates of the Tg197 mice in the *in vivo* long-term efficacy study. The percent survival rates of animals injected with antibody or vehicle twice weekly starting at 3 weeks of age were: vehicle-treated group = 80% (euthanized at week 11), 1 mg/kg adalimumab group = 50% (euthanized at week 16), and 1 mg/kg ozoralizumab-treated group = 100% (euthanized at week 16). Survival curves were compared using the log-rank test. **(B)** Changes in body weight at the time periods indicated. Results are expressed as the mean ± SEM (n = 5–10).

Supplemental Figure 2

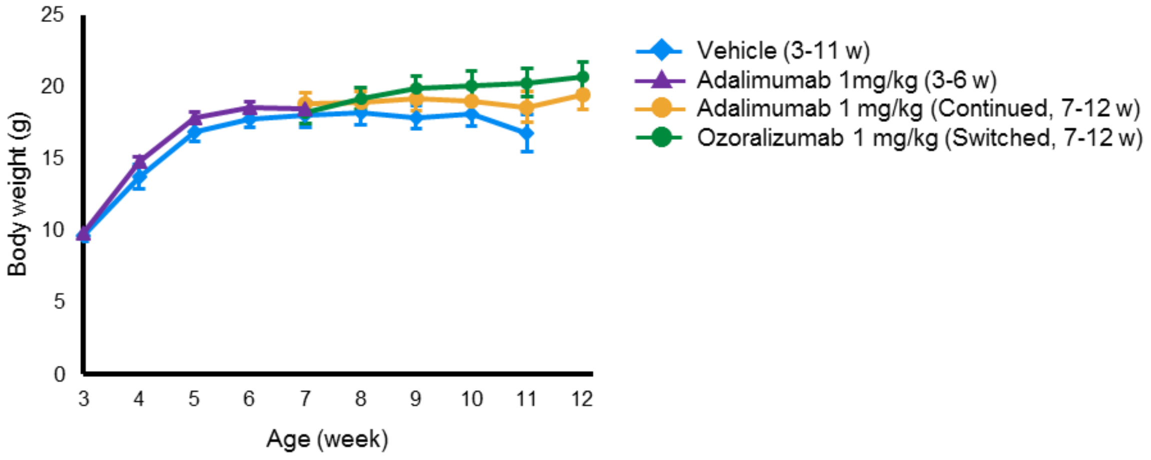

Supplementary Figure 2. Changes in body weight of the Tg197 mice during administration of adalimumab or ozoralizumab in the second study. Body weights were monitored weekly from 3 weeks of age to 11 or 12 weeks of age. Results are expressed as the mean ± SEM (n = 10–32).
